# HLA-E circulating and genetic determinants in schizophrenia and bipolar disorder

**DOI:** 10.1038/s41598-021-99732-9

**Published:** 2021-10-12

**Authors:** Wahid Boukouaci, Mohamed Lajnef, Jean-Romain Richard, Ching-Lien Wu, Jihène Bouassida, Ismail Rafik, Marianne Foiselle, Céline Straczek, Esma Mezouad, Soumia Naamoune, Sofiane Salah, Mohamed Amin Bencharif, Arij Ben Chaaben, Caroline Barau, Philippe Le Corvoisier, Marion Leboyer, Ryad Tamouza

**Affiliations:** 1 INSERM, IMRB, Translational Neuropsychiatry, AP-HP, DMU IMPACT, Fédération Hospitalo-Universitaire de Médecine de Précision en Psychiatrie (FHU ADAPT), Univ Paris Est Créteil, Fondation FondaMental, 94010 Créteil, France; 2 Pharmacie Hospitalière, HU Henri Mondor, 94010 Créteil, France; 3 Plateforme de Ressources Biologiques, AP-HP, HU Henri Mondor, 94010 Créteil, France; 4 Inserm, Centre d’Investigation Clinique 1430 et AP-HP, Hôpitaux Universitaires Henri Mondor, Univ Paris Est Creteil, 94010 Créteil, France; 5 Département Hospitalo-Universitaire de Psychiatrie, Hôpital Albert Chenevier, 40 rue de Mesly, 94000 Créteil, France

**Keywords:** Immunological techniques, Diagnostic markers, Predictive markers, Biological techniques, Adaptive immunity, Immunogenetics, Inflammation, Translational immunology

## Abstract

Schizophrenia (SZ) and bipolar disorders (BD) are severe mental illnesses that lack reliable biomarkers to guide diagnosis and management. As immune dysregulation is associated with these disorders, we utilized the immunoregulatory functions of the natural killer cell inhibitory HLA-E locus to investigate the relationships between HLA-E genetic and expression diversities with SZ and BD risk and severity. Four hundred and forty-four patients meeting DSM-IV criteria for SZ (N = 161) or BD (N = 283) were compared to 160 heathy controls (HC). Circulating levels of the soluble isoform of HLA-E molecules (sHLA-E) were measured and HLA-E*01:01 and HLA-E*01:03 variants genotyped in the whole sample. sHLA-E circulating levels were significantly higher in both SZ and in BD patients compared to HC (pc < 0.0001 and pc = 0.0007 for SZ and BD, respectively). High sHLA-E levels were also observed in stable SZ patients and in acute BD patients experiencing depressive episodes when comparisons were made between the acute and stable subgroups of each disorder. sHLA-E levels linearly increased along HLA-E genotypes (*p* = 0.0036). In conclusion, HLA-E variants and level may have utility as diagnostic biomarkers of SZ and BD. The possible roles of HLA diversity in SZ and BD etiology and pathophysiology are discussed.

## Introduction

Schizophrenia (SZ) and Bipolar Disorders (BD) are complex groups of severe and chronic psychiatric disorders associated with reduced psychosocial functioning and with an approximately 20 year decrease in life expectancy^1–3^. Their management still relies on clinical assessments as there are no biomarkers to guide diagnosis, prognosis, or disease course. A broad body of replicated data indicates that immune dysfunction contributes to the pathogenesis of both disorders, at least for a significant subset of patients. This provides a direction to clarify disease pathophysiology and novel treatment targets^4^. Dysregulated immunity is believed to arise from complex interactions of genes and the environment, involving both innate and adaptive immunity. This can lead to chronic inflammation and autoimmunity, two intertwined processes that can implicate the human leucocyte antigen (HLA) system^5^. Hosted by the major histocompatibility complex (MHC), the HLA system encodes molecules pivotal for mounting pro-inflammatory processes. This can arise due to the extreme polymorphism of classical HLA alleles that condition antigen-presentation function. The HLA system is also characterized by immuno-modulatory properties essential for the homeostatic control of a given inflammatory episode and mediated by the HLA non classical molecules which are encoded by four loci, namely HLA-E, -F, -G, and -H^6^.

The MHC/HLA region has been repeatedly shown to be relevant to SZ and BD pathophysiology by a number of experimental approaches, including genome wide association studies (GWAS)^7–10^, HLA-imputation methods^11^ and HLA-haplotype-based studies^12,13^. Unlike the classical HLA alleles which are characterized by an extreme rate of polymorphism, the HLA non classical ones are almost pauci- or monomorphic and therefore provide a means to analyze the role of HLA in human health and disease^14^.

In particular, HLA-E are cell surface molecules ubiquitously expressed and encoded by only two functional alleles, HLA-E*01:01 and HLA-E*01:03, which are distributed at equal frequencies worldwide due to an evolutionary balancing selective process^15^. The HLA-E*01:01 and HLA-E*01:03 alleles encode molecules that are differentially expressed at the cell surface with the former almost undetectable while the latter is more highly and appropriately expressed^16,17^. HLA-E molecules also exist as a soluble circulating isoform, soluble HLA-E (sHLA-E), which result from the shedding of membrane bound HLA-E molecules induced by stressful events, such as infections and/or inflammation^18^.

Upon binding to self-peptides from various HLA-class I molecules, HLA-E molecules modulate NK cell responses through interaction with the CD94-NKG2A inhibitory NK cell receptor. The main consequence of this interaction is the inhibition of the NK cell-mediated cytotoxicity and cytokine production^16,19,20^. HLA-E molecules also play a role in the binding of microbial derived-peptides from human pathogens (viruses and bacteria), with consequent induction of T cell responses^21^.

Given such pivotal functions, especially in NK-cell-mediated immuno-surveillance, the HLA-E loci has been extensively studied both at genetic and/or expression levels in various immune-related pathologies, including infections, inflammation and autoimmunity^22^. However, the HLA-E loci has been little investigated in psychiatric conditions, despite the intimate association of psychiatric conditions with comorbid immune dysregulation including genetically determined inefficient anti-infectious responses, chronic inflammation and auto-immunity^23^.

Consequently, we performed a case control study involving 161 SZ patients, 283 BD patients and 160 healthy controls (HC) in order to analyze: (i) the circulating levels of the sHLA-E isoform and its correlation with disease risk and patients clinical characteristics; and (ii) the distribution of the HLA-E*01:01 and HLA-E*01:03 genotypes and their potential correlation with sHLA-E levels.

## Results

Demographic and clinical characteristics of the study participants are shown in Table 1.

**Table 1:** Demographic and clinical characteristics of the study subjects.

Variable	BD (n = 283)	SZ (n = 161)	HC (n = 160)	Overall *p* value
sHLA-E (pg/ml): (mean ± SD)	260.9 ± 240.9	316.6 ± 215.6	189.3 ± 143.2	< 0.001
Age	40.5 ± 14.6	37.4 ± 11.4	35.6 ± 13.2	0.003
Sex: F (%)	53.0	31.5	51.9	< 0.001
Acute status (%)	60.8	58.0	–	0.486
Early onset (%)	44.2	18.3	–	< 0.001
Age of first episode (mean ± SD)	25.0 ± 9.7	23.4 ± 6.5	–	0.526
PPANSS (mean ± SD)	15.9 ± 8.0	18.5 ± 6.7	–	< 0.001
NPANSS (mean ± SD)	11.8 ± 4.7	19.9 ± 7.3	–	< 0.001
GPANSS (mean ± SD)	29.3 ± 8.3	35.6 ± 9.0	–	< 0.001
TPANSS (mean ± SD)	57.1 ± 17.0	74.2 ± 19.1	–	< 0.001
TMADRS (mean ± SD)	13.1 ± 10.4	12.2 ± 8.0	–	0.984
TYMRS (mean ± SD)	12.5 ± 11.2	8.9 ± 9.0	–	0.001
CGI (mean ± SD)	4.3 ± 1.4	4.7 ± 1.4	–	0.507
GAF-SYMP (mean ± SD)	47.2 ± 19.9	40.5 ± 19.5	–	< 0.001
GAF-HANDICAP (mean ± SD)	47.4 ± 15.9	37 ± 12.3	–	< 0.001
TCDSS (mean ± SD)	4.6 ± 5.8	3.5 ± 4.3	–	0.507

Psychotic symptoms were assessed using the positive and negative syndrome scale (PANSS); positive PANSS (PPANSS); negative PANSS (NPANSS); Global PANSS (GPANSS); Total PANSS (TPANSS). All patients were evaluated for mania with the Total Young Mania Rating Scale (TYMRS). Depression was scored using the Total Calgary Depression Scale (TCDSS) and the Total Montgomery-Asberg Depression Rating Scale (TMADRS). General functioning was assessed with the Global Assessment of Functioning Scale (GAF); GAF symptom score (GAF-SYMP) and GAF Handicap score (GAF-HANDICAP).

The total sample included 161 SZ patients, 283 BD patients and 160 healthy controls (HC). Female patients comprised 31.48% and 52.98% of SZ and BD patients respectively. The mean ages at inclusion were 37.4 (± 11.4) years and 40.5 (± 14.6) years for SZ and BD patients. The age of the first episode was 23.4 (± 6.5) years for SZ and 25.0 (± 9.66) years for BD patients, of which 18.25% and 44.19% had an early-onset of their psychiatric disorder (≤ 18 years and ≤ 22 years for SZ and BD respectively). The mean age of controls was 35.6 (± 13.2) years, being comprised of 51.88% females.

**Figure 1:**
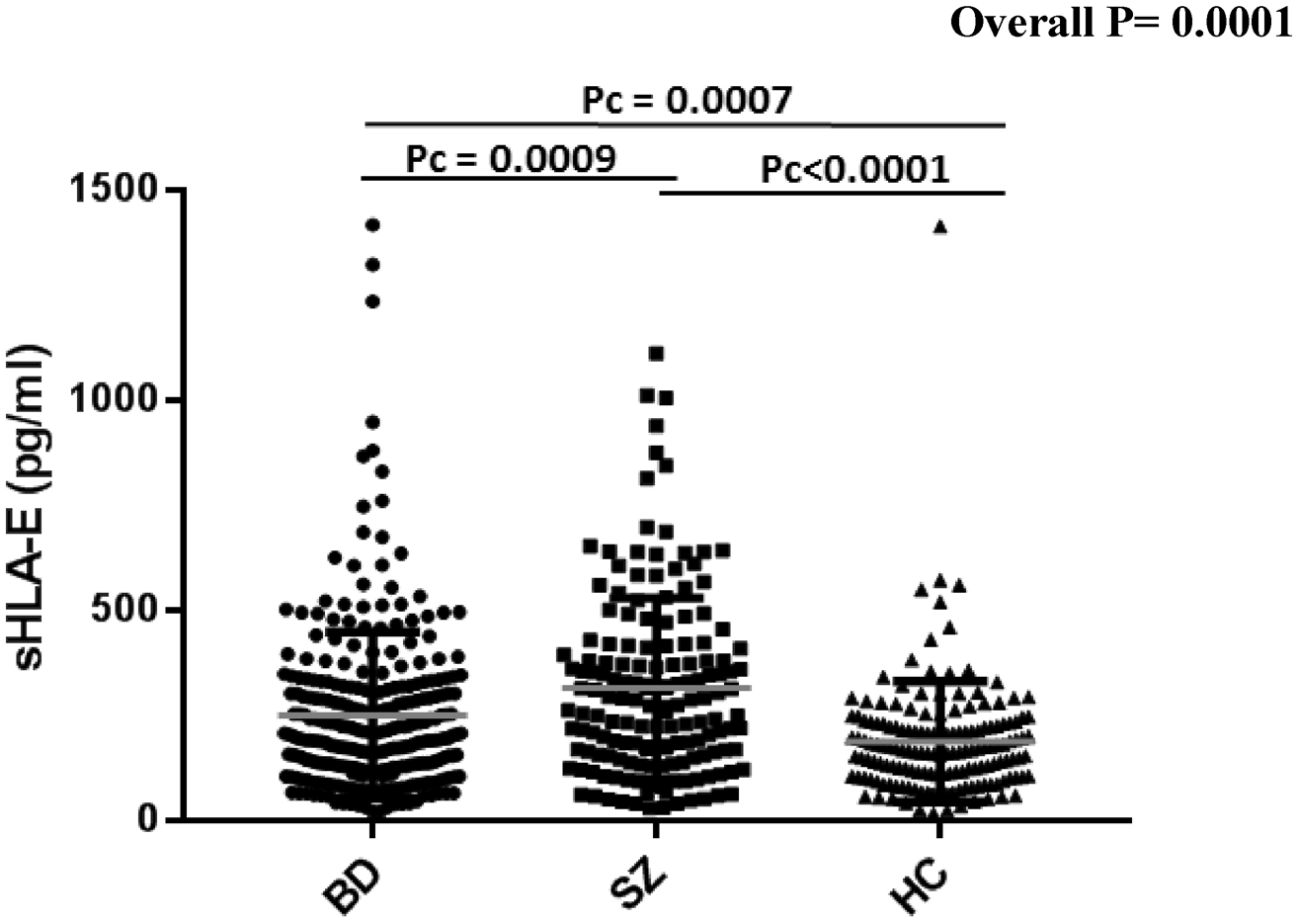
Analysis of the distribution of circulating sHLA-E levels among SZ, BD patients and healthy controls. Statistically significant increase in both patient subgroups (mean sHLA-E: 316.6 vs. 189.3, pc < 0.0001 in SZ vs. HC and 260.9 vs. 189.3, pc = 0.0007 in BD vs. HC) and between SZ and BD patients (mean sHLA-E: 316.6 vs. 260.9, pc = 0.0009 in SZ vs. BD). *p* value corrected using the Bonferroni test.

**Figure 2:**
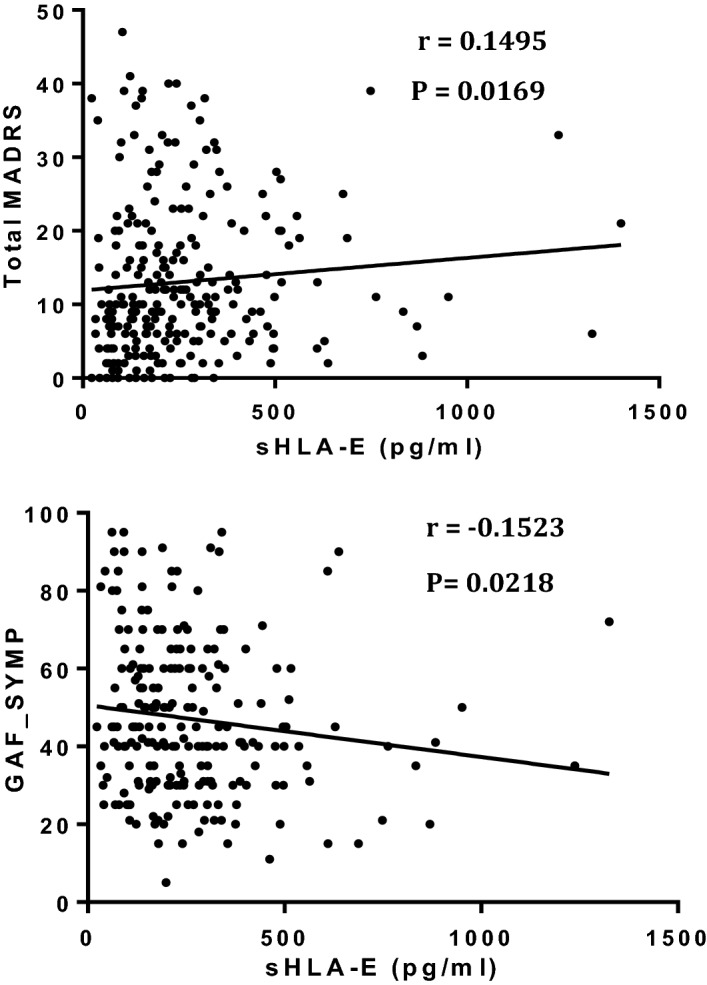
Relationship between circulating levels of sHLA-E, depressive scores measured by MADRS and functioning measured by GAF scores (*p* = 0.017 and *p* = 0.022 respectively).

**Figure 3:**
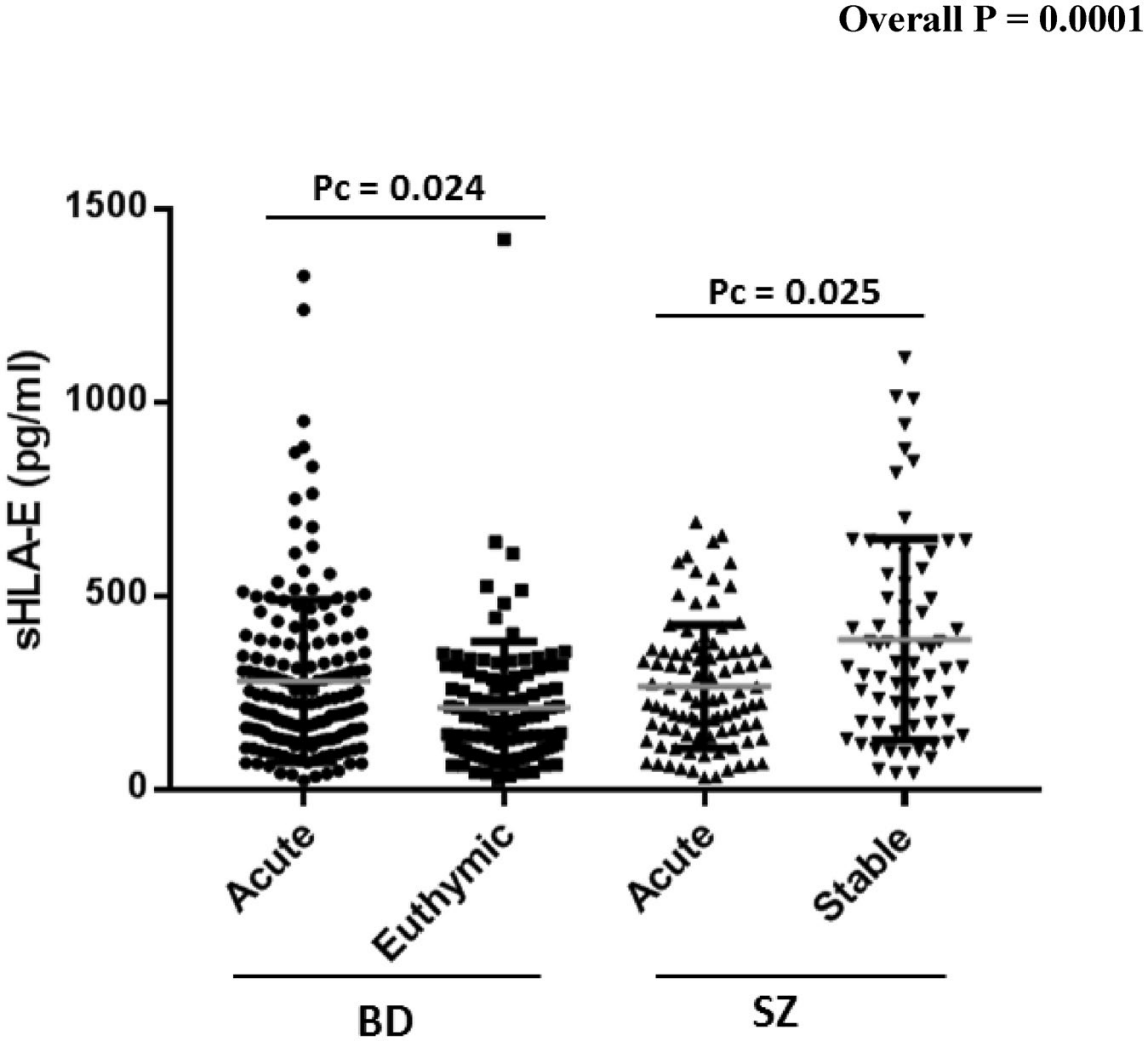
Analysis of sHLA-E distribution according to acute and stable phase of SZ or BD (265.14 vs. 387.05, pc = 0.025 in acute and stable SZ patients respectively) and (278.9 vs. 232.48, pc = 0.024 in acute BD episode and euthymic phase respectively). *p* value was corrected using the Bonferroni test.

The analysis of the distribution of circulating sHLA-E levels among SZ and BD patients revealed a statistically significant increase in both patient subgroups as compared to HC (mean sHLA-E: 316.6 vs. 189.3, pc < 0.0001 in SZ vs. HC and 260.9 vs. 189.3, pc = 0.0007 in BD vs. HC) (Fig. 1). Notably, the two patient sub-groups differed from each other regarding sHLA-E levels (mean sHLA-E: 316.6 vs. 260.9, pc = 0.0009 in SZ and BD patients, respectively), (Fig. 1). We then looked for potential correlations between sHLA-E circulating levels and symptomatic characteristics of patients. In BD patients, we found that high levels of sHLA-E were positively associated with high scores of depression as measured by the MADRS and low scores of global functioning as assessed by the GAF scale (*p* = 0.017 and *p* = 0.022 respectively) (Fig. 2), suggesting that high sHLA-E may characterize patients with a severe acute depressive episode. No association was observed between sHLA-E levels and any of the assessed psychotic or mood symptoms in SZ patients. Stratifying patients according to their disease status (acute episode or stable phase), we observed that the mean sHLA-E was higher in stable, versus acute, episode SZ patients (387.05 vs. 265.14, pc = 0.025 in stable and acute SZ patients respectively) while, conversely in BD patients, the mean sHLA-E was higher in patients having an acute, versus stable, episode (278.9 vs. 232.48, pc = 0.024 in acute mood episode and stable phase respectively) (Fig. 3). Finally, the logistic regression evaluating the potential impact of sex, age and BMI on sHLA-E levels showed that only age negatively correlated with sHLA-E levels (*p* = 0.006), Table 2.

**Table 2:** Linear logistic regression.

Predictors	Log sHLA-E		
	Estimates	CI	*p*
Age	− 0.0092	− 0.0157 to − 0.0027	**0.006**
BMI	0.0112	− 0.0033 to 0.0257	0.131
Sex [M]	0.0549	− 0.1082 to 0.2180	0.508

Bold value indicates statistically significant.

Adjusted R^2^/0.034/0.024.

*CI* confidence interval, *p p* value.

**Figure 4:**
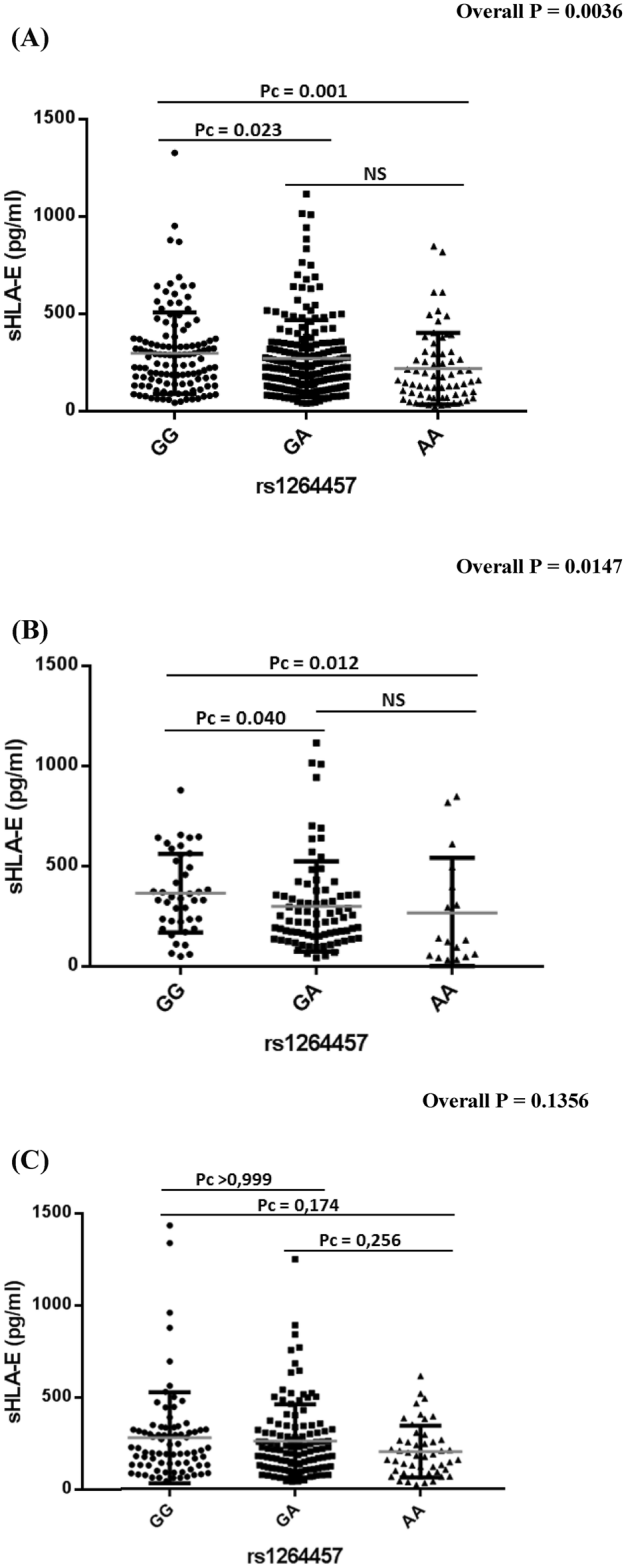
Analysis of sHLA-E levels according to the genetic distribution of the HLA-E *rs*1264457 AA, AG and GG genotypes in the whole cohort of patients and in the subgroup of SZ patients. (**A**) In the whole cohort of patients including SZ and BD, sHLA-E levels increase in a linear manner along HLA-E genotypes (AA = 218.5, AG = 270.27, GG = 315.28, overall *p* = 0.0036). (**B**) In patients with SZ, sHLA-E levels are also observed to increase along HLA-E genotypes (AA = 265.65, AG = 298, GG = 364.58, overall *p* = 0.0147). (**C**) In patients with BD, no difference was observed concerned the distribution of sHLA-E according to the HLA-E *rs*1264457 AA, AG and GG genotypes (AA = 202.1, AG = 259.9, GG = 277.1, overall *p* = 0.1356). All figures were generated using GraphPad Prism version 7.00 for Windows, GraphPad Software, La Jolla California USA, www.graphpad.com.

In order to test if the observed high production of sHLA-E is under genetic control, we analyzed the potential associations between the HLA-E *rs*1264457 genotypes, that categorize HLA-E alleles into HLA-E*01:01 and HLA-E*01:03 specificities, and circulating sHLA-E levels. In the whole cohort of patients, we found that sHLA-E levels increase in a linear manner along HLA-E genotypes (AA = 218.5, AG = 270.27, GG = 315.28, overall *p* = 0.0036) (Fig. 4A). Such correlation was also observed when patients and HC were pooled (overall *p* = 0.008) (data not shown). Similar distribution of sHLA-E levels according to HLA-E genotypes (AA = 265.65, AG = 298, GG = 364.58, overall *p* = 0.0147) was observed in patients with SZ (Fig. 4B), although not in the BD patient sub-group (Fig. 4C) (AA = 202.1, AG = 259.9, GG = 277.1, Overall *p* = 0.1356).

## Discussion

HLA-E molecules by interacting with the NK cell inhibitory NKG2A receptor are major players of NK cell mediated immuno-surveillance by preventing inappropriate or unwanted NK cell activation against self. To a lesser extent, HLA-E, by presenting pathogen derived antigens to T cells, partly participates in the induction of T lymphocyte-mediated adaptive immune responses^21^.

Here, we first observed that circulating sHLA-E levels were significantly higher in SZ and in BD patients as compared to HC, a finding that could implicate the functional properties of the soluble form of the HLA-E molecule in SZ and BD.

sHLA-E, similarly to other soluble isoforms of cell-surface expressed molecules, results from the metalloproteinase-mediated shedding of the membrane-bound molecules especially during stressful events, such as inflammation, which enhances their cell-surface expression. sHLA-E isoforms display immuno-regulatory properties similar to that of the membrane-bound molecules^18^. Accordingly, high circulating levels of sHLA-E were observed in various pathological contexts schematically including highly inter-twinned pathophysiological processes such as infection, inflammation and neoplasic transformation. For example, high levels of sHLA-E were observed both in periphery and in the cerebrospinal fluid (CSF) of relapsing–remitting multiple sclerosis (MS) patients as compared to non-MS, non-inflammatory neurological controls, although similar to that observed in non-MS inflammatory neurological patients. Such data indicate that sHLA-E likely reflects inflammatory processes rather than MS per se. However, this study also showed that among MS patients, sHLA-E levels were higher in the stabilized, versus active, group indicating their protective status against active MS^24^. In Takayashu arteritis which is characterized by arterial wall inflammation, sHLA-E levels are higher in the active, versus stabilized, phase of the disease, indicating that high sHLA-E levels also reflect active inflammatory processes^25^. In Kawasaki disease, another systemic inflammatory vasculitis, high sHLA-E levels are also observed in patients, versus controls^26^. The Japanese encephalitis virus, a flavivirus responsible for encephalitis in Asian children, upregulates HLA-E cell-surface levels, coupled to an enhanced release of sHLA-E, suggesting a potential immuno-evasive strategy likely developed by the viral agent against host mediated immune responses^27^. In cancer, whilst high sHLA-E levels associate with a better overall survival in neuroblastoma^28^, high sHLA-E levels were also observed: (i) in advanced stages of chronic lymphocytic leukemia, with sHLA-E levels decreasing after treatment^29^; and (ii) in patients suffering from acute leukemia, as compared to healthy controls^30^. Finally, in melanoma, several studies show high sHLA-E levels to be released by neo-transformed melanocytes^31^, a finding at the origin of the recent development of a novel immune check point inhibitor targeting the HLA-E specific NKG2A inhibitory receptor, namely Monalizumab^32^.

Overall, such data highlight the role of sHLA-E in an array of diverse medical conditions where the pathophysiology is intimately linked to inflammatory conditions and alterations in patterned immune responses. Generally, high sHLA-E levels may be beneficial by alleviating inflammatory processes and deleterious by promoting tumor evasion and immune escape during infection.

Interestingly, the present data show high sHLA-E levels to be mainly evident in BD patients experiencing acute and severe depressive episodes and, conversely, in stabilized SZ patients. Such data indicate a possible differential role of NK cells, and their regulation, in these two disorders, plausibly mediated by a sHLA-E-mediated NK cell inhibition being deleterious in BD, whilst being beneficial in SZ. Such observations in BD may be parsimonious with previous data showing a role for NK cells in depression. Early studies showed depression to be reliably associated with the suppression of mitogen-induced lymphocyte proliferation and with a reduced NK activity^33^, with NK cells being sensitive to stress responses that compromise their functions in depressed patients^34^. Depression may be significantly associated with other medical conditions classically linked to variations in NK function, including infection following coronary artery bypass grafting, where female patients who experience major depression had lower NK cell cytotoxicity, more self-reported illness and more infectious events as compared to those without depressive symptoms^35^. More recently, a study analyzing correlations between circulating lymphocytes subsets and Diffusion Tensor Imaging measures of water diffusion, fMRI corticolimbic functional response and connectivity in BD patients, showed a likely lithium mediated protective effect of NK cell subsets against alteration of white matter microstructure and functional connectivity^36^. Furthermore, we recently analyzed NK cell functions in first episode psychosis (FEP) patients who latter developed either BD or SZ and observed an overall impairment of NK cell functional properties. More specifically, NK cells from patients who developed BD showed an inability to produce IFN-γ, a cytokine pivotal to NK function, while SZ-derived NK cells exhibit a suppressed capacity to mount cytotoxic responses in the presence of target cells^37^. These preliminary data may be parsimonious with the findings reported here as: (i) deficient IFN-γ production and altered patterned immune responses^38,39^ may contribute to the alterations observed in acute BD episodes; and (ii) the deficient NK cytotoxic functions may alleviate inflammatory processes in stabilized SZ.

Overall, such observations highlight the importance of NK cells in major mood and psychotic disorders, with a possible role for sHLA-E regulation of NK cells on different aspects of BD and SZ pathophysiology.

This is supported by several studies that have evaluated the relationship of SZ and BD with the genetic and expression diversities of HLA-G, another potent HLA-non classical immune modulator via NK cell inhibition^40^. These studies showed: (i) that genetically determined low expression of the tolerogenic HLA-G molecules was associated with SZ severity implying that high level of HLA-G likely confers a protective effect against severe forms of SZ via NK inhibition^41–44^, as with sHLA-E in the current study; and (ii) that genetically determined HLA-G low expression confers protection against BD^45,46^, whilst high HLA-G levels increases BD risk, which is also parsimonious with the results of the present study. However, it is important to note that the role of NKG2A and other inhibitory NK cell receptors is complicated by some paradoxical effects on NK cell function, including inhibiting NK effector responses against a target cell, whilst also improving/educating their functional competence for a given individual, a phenomenon called licensing^47^.

The classical HLA alleles-mediated adaptive immune responses have long been associated with inflammatory responses, including the inflammatory HLA 8.1 ancestral haplotype in BD and in SZ. Although we found associations between the HLA 8.1 AH and severe forms of the BD, i.e. rapid cycling and suicidal behavior^12^, in SZ we observed that the HLA 8.1 AH confers protection or delays age of disease onset^6,13^. Given that the HLA cluster is pivotal to both immune processes and brain development, these results suggest that HLA-linked processes may be implicated in mechanisms involved in ontogenic and neurodevelopmental difference between SZ and in BD.

Finally, we also observed that the HLA-E genetic dimorphism influence sHLA-E levels, the highly expressed HLA-E*01:03 allele is associated with enhanced levels of the soluble isoform in SZ and the whole patient sample, but strikingly not in BD alone. Again, this may indicate the differential impact of HLA genetics in SZ and BD. This may contrast with the data showing the two HLA-E alleles to have equally distributions worldwide, as both are required for physiological processes.

In the present study we have to acknowledge that the potential impact of psychotropic drugs known to exhibit anti-inflammatory properties was not analyzed because of the non-availability of exhaustive information on patient treatments. Indeed, it would have been interesting to assess the potential influence of therapeutic compounds used in the management of BD and SZ. Even if they target different pathways, some of these drugs are characterized by potent anti-inflammatory properties. For example Lithium (Li) inhibits glycogen synthetase kinase-3-beta (GSK-3β), thereby decreasing levels of pro-inflammatory cytokines, including tumor necrosis factor alpha and interleukin 6 (IL-6). Li also promote the synthesis of anti-inflammatory molecules such as IL-2 and IL-10 and modulates glial cellular inflammation^48,49^. Commonly, SZ treatment is provided by second-generation antipsychotics, which also have antioxidant and anti-inflammatory effects^50,51^, with the high levels of pro-inflammatory cytokines observed in first episode patients suppressed following the initiation of anti-psychotic therapy^52^.

We have also to acknowledge the absence of patient follow up, thereby preventing us from following up sHLA-E dynamics over the course of transitions between acute and stable states, and the non-availability of data concerning peripheral inflammatory markers that might reflect different sHLA-E level status.

Overall, the data of the current study show the relevance of sHLA-E to the pathophysiology of acute and stable phases of BD and SZ, including via the genetic determinant of sHLA-E circulating levels. The data also has relevance as to the differential and overlapping etiology and pathophysiology of BD and SZ. Future longitudinal studies are warranted to follow sHLA-E evolution during transition between acute and stabilized phases of the two disorders. It will also be important to link sHLA-E and other HLA alleles to wider data on SZ and BD pathophysiology, including alterations in the gut microbiome and suboptimal mitochondrial function and the treatment implications arising therefrom.

## Material and methods

### Study subjects

Patients with SZ (n = 161) and BD (n = 283) were systematically included from the university affiliated psychiatric department of the Henri Mondor Hospital (Créteil, France). They were included either during hospitalization for an acute mood or psychotic episode or during a visit at a day care hospital during a stable phase of their disease. They were compared to 160 healthy controls (HC), recruited in the clinical investigation center (CIC) of Henri Mondor Hospital (Créteil). Blood drawing for patients and control was done around 10am without requiring fasting. Patient diagnosis were established using the French version of the Structured Clinical Interview (SCID) for DSM-IV^53^ while HC were assessed using the French version of the Diagnostic Interview for Genetic Studies (DIGS)^54^. All patients were evaluated for mania with the Young Mania Rating Scale (YMRS)^55^. Depression was scored using the Calgary Depression Scale (CDSS) for SZ^56^ and the Montgomery-Asberg Depression Rating Scale (MADRS)^57^ for BD. Psychotic symptoms were assessed using the positive and negative syndrome scale (PANSS)^58^. General functioning was assessed with the Global Assessment of Functioning Scale (GAF)^59^. Subjects who scored MADRS above 15, YMRS above 8 and PANSS above 60 were considered to be in an acute episode. Other characteristics including age at inclusion and age at disease onset, gender, duration of untreated psychosis and characteristic of first episode were recorded. All participants were carefully interviewed by trained psychiatrists or psychologist, with blood samples then taken and sent to the Biological Research repository of the Henri Mondor University Hospital for immediate processing and storage until experimental analysis. All subjects gave written informed consent for their participation to the study, which was approved by the Comité de Protection des Personnes. Ile-de-France III.

All methods were performed in accordance with the relevant guidelines and regulations.

### Soluble HLA-E evaluation and HLA-E genotyping

Circulating plasma levels of sHLA-E were determined using commercially available ELISA kits (Cloud-Clone Corp, Kathy, Texas, USA) according to the manufacturer’s instructions. All samples were tested in triplicates.

Genomic DNA was extracted from EDTA-treated peripheral blood samples using the automated Maxwell DNA purification system (Promega, USA) and quantified by mean of the Quant-iT™ PicoGreen^®^ dsDNA Assay Kit (Thermo Fisher Scientific, USA). HLA-E genotyping was performed by a TaqMan^®^ 5′-nuclease assay (Applied Biosystems, Foster city, CA, USA) with allele-specific fluorogenic oligonucleotide probes for HLA-E alleles i.e. HLA-E*01:01 and HLA-E*01:03 allowing the discrimination of the genotypes of each studied pair of alleles. The primer and probe sequences were designed by assay on demand (Applied Biosystems, Foster city, CA, USA).

### Statistical analysis

Demographic characteristics of the bipolar, schizophrenia and control groups were compared using chi squared (X^2^) test for categorical variables and Wilcoxon signed-rank or Student test for continuous variables. As the soluble isoform of HLA-E molecules (sHLA-E) scores did not follow a normal distribution (as shown by Shapiro tests with a *p* value < 0.05, and graph), the correlations between sHLA-E with the functioning and depression (GAF and MADRS) scores were first assessed using Spearman’s correlation coefficient followed by a suitable significance test. Subsequently, the Wilcoxon signed-rank and Kruskal–Wallis rank sum tests were performed to assess the associations between sHLA-E and the categorical variables (diagnosis, namely BD, SZ, HC) and disease phase (acute and stabilized). A linear regression model was used to evaluate relationships between age, gender, BMI as independent variables and a sHLA-E level as a continuous dependent variable. Significance was defined as *p* < 0.05 for all statistical tests, which were performed using R software R version 4.0.3 and Graphpad PRISM 9 to generate graphs. *p* values were corrected using the Bonferroni method when needed and then were designated as corrected *p* value (pc).

## Data Availability

All the data generated in the present study are fully available.
